# Prevalence of temporomandibular joint dysfunction in patients with ankylosing spondylitis and comparison of the findings with healthy controls

**DOI:** 10.1590/1806-9282.20240807

**Published:** 2024-09-30

**Authors:** Büşra Şirin Ahısha, Fatma Nur Kesiktaş

**Affiliations:** 1 University of Health Sciences, Istanbul Physical Medicine and Rehabilitation Hospital – İstanbul, Turkey.

**Keywords:** Ankylosing spondylitis, Temporomandibular joint disorders, Temporomandibular disorders, TMJ, Spondylarthritis

## Abstract

**OBJECTIVE:**

This study aimed to assess the prevalence of temporomandibular dysfunction in ankylosing spondylitis patients and healthy controls, examining the relationship between temporomandibular dysfunction and disease activity in ankylosing spondylitis patients, as well as associations with psychosocial factors.

**METHODS:**

The study included 113 ankylosing spondylitis patients and 110 healthy individuals aged 18–75. Temporomandibular dysfunction presence was evaluated using Diagnostic Criteria for Temporomandibular Disorders Axis I. Disease activity was assessed with the Bath Ankylosing Spondylitis Disease Activity Index, Bath Ankylosing Spondylitis Metrology Index, and Bath Ankylosing Spondylitis Functional Index.

**RESULTS:**

Among healthy individuals, 60.9% did not receive a temporomandibular dysfunction diagnosis, while 39.1% received at least one diagnosis. In contrast, 69.9% of the 113 ankylosing spondylitis patients received at least one temporomandibular dysfunction diagnosis, and only 30.1% were not included in any diagnosis group (p<0.001). Joint (p=0.001) and pain disorders (p=0.008) were significantly more common in the ankylosing spondylitis group than in the healthy controls. Significant associations emerged between Bath Ankylosing Spondylitis Disease Activity Index (p<0.001) and Bath Ankylosing Spondylitis Functional Index (p=0.005) scores and pain disorders.

**CONCLUSION:**

Temporomandibular dysfunction is more prevalent in ankylosing spondylitis patients than in healthy individuals, linked to increased joint issues and pain associated with disease activity.

**ClinicalTrials.gov ID:**

NCT05839925.

## INTRODUCTION

Ankylosing spondylitis (AS), known as the prototype of the spondyloarthropathy group, is a chronic inflammatory rheumatic disease^1^. Symptoms typically manifest in the second or third decade of life. It occurs approximately twice as often and with more severe outcomes in males^2^. While axial involvement is common, there is also involvement in peripheral joints and extra-articular organs. In AS, it is believed that peripheral joint involvement and synovitis, one example of which is temporomandibular joint (TMJ) involvement, can occur^3^. The exact mechanism of TMJ involvement in AS is not fully understood, but mechanisms such as destruction in the joint capsule or disc, synovitis development resulting from damage to articular surfaces, and cranio-cervical posture changes have been suggested. Inflammation occurring in the capsule and disc leads to functional impairment, which in turn causes degenerative changes in the later stages^4^. Inflammation, erosion, and new bone formation in entheses areas secondary to mechanical loading in the TMJ are implicated in disc displacement^5^. It is known that TNF-alpha and IL-17 are cytokines with a common role in TMJ synovitis development and AS pathogenesis. This suggests that TMJ involvement could be one of the clinical manifestations of AS^3^.

Therefore, this study aimed to determine the frequency of temporomandibular dysfunction (TMD) in patients diagnosed with AS, evaluate its relationship with disease activity, and compare these findings with healthy controls.

## METHODS

Our research was conducted in a cross-sectional clinical study design. The research ethics committee approval (protocol no: 2022/98) was obtained from the Bakırköy Dr. Sadi Konuk Training and Research Hospital Clinical Research Ethics Committee. Informed consent was obtained from the participants before the study began. The study was conducted following the principles of the Helsinki Declaration.

A total of 113 consecutive patients aged 18–75 years who applied to the outpatient clinics of the Health Sciences University Istanbul Physical Medicine and Rehabilitation Training and Research Hospital between April 1, 2022, and April 1, 2023, and who were diagnosed with AS according to the Modified New York Criteria, and 113 healthy volunteers aged 18–75 years were included in the study. Ultimately, three healthy individuals withdrew. Exclusion criteria for our research included changes in AS treatment in the last 6 months, preexisting pathology related to the TMJ and associated structures before AS diagnosis, use of medications affecting bone metabolism, neurological or cognitive deficits, history of trauma, malignancy, infection, or surgery in the head and neck region, dental or periodontal pain, and a history of orthodontic treatment.

### Study design

Demographic data were recorded. We assessed the presence of TMD in patients using the Diagnostic Criteria for Temporomandibular Disorders (DC/TMD) Axis I, which were published in 2014 and translated into Turkish in 2016^6^.

To assess mobility and vertebral limitations in AS patients, the Bath Ankylosing Spondylitis Metrology Index (BASMI)^7^ was used, consisting of measurements such as tragus-wall distance, intermalleolar distance, modified Schober test, lateral flexion, and cervical rotation. The Bath Ankylosing Spondylitis Disease Activity Index (BASDAI)^8^ was utilized to evaluate disease activity, and the Bath Ankylosing Spondylitis Functional Index (BASFI)^9^ was employed to assess the functional status.

### Statistical analysis

The Shapiro-Wilk test indicated that the data did not follow a normal distribution. Therefore, quantitative variables were summarized using median, minimum, and maximum values, while qualitative variables were described using frequencies and percentages. The chi-square test was used to compare qualitative variables, and the Mann-Whitney U test was applied to compare quantitative variables with qualitative variables in two categories. A type I error rate of 0.05 was considered. Data analysis was performed using IBM SPSS 25.

## RESULTS

In this study, pain-free opening (p=0.023), maximum assisted opening (p=0.042), and maximum unassisted opening (p=0.003) values were significantly lower in the AS group compared to the healthy group. There were no significant differences observed between the two groups in terms of protrusion (p=0.621), right lateral movement (p=0.598), left lateral movement (p=0.091), and total lateral movement (p=0.184) measurement values (Table 1).

**Table 1:** Comparison of surviving and exitus groups, discharged and hospitalized groups based on injury severity score, inferior vena cava collapsibility index, and Edmonton Frail Scale scores.

	Healthy group Median (min–max)	Ankylosing spondylitis Median (min–max)	p-value
Pain-free opening*	45 (28–65)	42 (22–55)	0.023
Maximum assisted opening*	51 (32–73)	49 (33–69)	0.042
Maximum unassisted opening*	50 (38–72)	47 (30–67)	0.003
Protrusion*	6 (2–10)	6 (0–15)	0.621
Right lateral*	9 (3–15)	8 (3–15)	0.598
Left lateral*	7.5 (2–15)	7 (1–15)	0.091
Total lateral*	17 (7–28)	16 (4–30)	0.184

*Median (min–max); Mann-Whitney U test. p<0.05 is considered significant. Statistically significant values are indicated in bold.

Among the 110 healthy individuals, 67 (60.9%) had no TMD diagnosis, while 43 (39.1%) were diagnosed with at least one TMD. Of the 113 AS patients, 79 (69.9%) had at least one TMD diagnosis and 34 (30.1%) had none. The difference between the groups was statistically significant (p<0.001) (Table 2).

**Table 2:** Comparison of participants’ temporomandibular joint examination findings and temporomandibular dysfunction diagnosis groups.

	Control n, (%)	AS n, (%)	p-value
TMD*			
No	67 (60.9)	34 (30.1)	<0.001
Yes	43 (39.1)	79 (69.9)	
Right TMJ disorders*			
No	91 (82.7)	68 (60.2)	<0.001
Disc displacement with reduction	17 (15.5)	27 (23.9)	
Disc displacement without reduction, without limited mouth opening	0 (0)	2 (1.8)	
Degenerative joint disease	2 (1.8)	16 (14.2)	
Left TMJ disorders*			
No	80 (72.7)	60 (53.1)	0.002
Disc displacement with reduction	24 (21.8)	34 (30.1)	
Disc displacement with reduction, with intermittent locking	1 (0.9)	0 (0)	
Disc displacement without reduction, without limited mouth opening	0 (0)	3 (2.7)	
Degenerative joint disease	5 (4.5)	16 (14.2)	
Myalgia*			
Yok	87 (79.1)	71 (62.8)	0.003
Local myalgia	22 (20)	31 (27.4)	
Myofascial pain (spreading or referral)	1 (0.9)	11 (9.7)	
TMJ pain*			
No	104 (94.5)	95 (84.1)	0.034
Right	1 (0.9)	1 (0.9)	
Left	1 (0.9)	2 (1.8)	
Bilateral	4 (3.6)	15 (13.3)	
Headache attributed to TMD*			
No	105 (95.5)	102 (90.3)	0.133
Yes	5 (4.5)	11 (9.7)	

*n (%), chi-square test. p<0.05 is considered significant. TMJ: temporomandibular joint, TMD: temporomandibular dysfunction, AS: ankylosing spondylitis. Statistically significant values are indicated in bold.

Out of 110 healthy individuals, 36 (32.7%) had joint disorders, compared to 61 of 113 AS patients (54%), with the AS group showing a significantly higher rate (p=0.001). The most common joint disorder in both groups was reduction disc displacement (Table 2). Among the 61 AS patients with joint disorders, 37 (60.7%) had bilateral and 24 (39.3%) had unilateral disorders, a statistically significant difference (p=0.019). In the healthy group, 86 of 110 individuals (78.2%) had no pain disorders, while 24 (21.8%) did. In the AS group, 43 of 113 patients (38.1%) had pain disorders, showing a significant difference (p=0.008). The presence of myalgia was significantly higher in the AS group compared to the healthy group (p=0.003). TMJ pain was also significantly more frequent in the AS group (p=0.034). Headache attributed to TMD was observed in 5 individuals (4.5%) in the healthy group and in 11 patients (9.7%) in the AS group, with no significant difference (p=0.133) (Table 2).

In the AS group, pain disorders were present in 19 of 67 male patients (28.4%) and 24 of 46 female patients (52.2%), with a significantly higher rate in females (p=0.010). No significant relationship was observed between pain disorders and age, marital status, smoking status, body mass index (BMI), duration of diagnosis, or age at symptom onset. Additionally, no statistically significant relationship was found between the presence of joint disorder and gender, age, marital status, smoking, BMI, human leukocyte antigen-B27 (HLA-B27) positivity, duration of diagnosis, or age at symptom onset.

In the AS patient group, a statistically significant relationship was observed between pain disorders and both BASDAI (p<0.001) and BASFI scores (p=0.005). However, the relationship between the BASMI total score and pain disorders was not significant (p=0.193). The diagnosis of joint disorders did not show a significant relationship with BASDAI (p=0.220), BASFI (p=0.979), or BASMI (p=0.911) scores (Table 3).

**Table 3:** The relationship between pain disorders and disease activity in ankylosing spondylitis patients.

	No pain disorders	Pain disorders	p-value
	Median (min–max)	Median (min–max)	
BASDAI*	4 (0–8)	6 (1–9)	<0.001
BASFI*	3.5 (0–9)	5 (0–10)	0.005
BASMI*	2 (1–7)	2 (0–8)	0.193
	**No joint disorders**	**Joint disorders**	**p-value**
	**Median (min–max)**	**Median (min–max)**	
BASDAI*	5 (0–9)	5 (1–9)	0.220
BASFI*	4 (0–9)	4 (0–10)	0.979
BASMI*	2 (1–7)	2 (0–8)	0.911

*Median (min–max), Mann-Whitney U test was used. p<0.05 is considered significant. Statistically significant values are indicated in bold.

## DISCUSSION

In this study, the prevalence of TMD evaluated using DC/TMD was found to be significantly higher in AS patients compared to the healthy group. Both pain-related and joint-related TMDs were more frequently observed in the AS patient group than in the healthy group.

The prevalence of TMD in the healthy population varies depending on the diagnostic algorithms used in studies; however, temporomandibular-related symptoms are observed in approximately 50% of adults^10^. In a study conducted in Finland, 100 prisoners were evaluated based on DC/TMD diagnostic criteria, and 76% of them were found to have joint disorders, while the rate of pain disorders was reported as 17%^11^. Out of 110 healthy volunteers, 67 (60.9%) had no TMD diagnosis, while 43 (39.1%) had at least one TMD. Among these, 36 (32.7%) had joint disorders and 24 (21.8%) had pain disorders. Specifically, 23 (20.9%) had muscle pain, 6 (5.4%) had TMJ pain, and 5 (4.5%) had TMD-related headaches. A review by Valesan et al.^12^ also examined the prevalence of TMD in the healthy population, reporting it to be approximately 31%. In another study using DC/TMD, 368 adults were examined and 60 patients (16.3%) were classified as having pain-related TMD, 48 patients (13%) had joint-related TMD, and 16 patients had both pain and joint-related TMD. Additionally, 1.6% were considered to have degenerative joint disease^13^. In our study, the rate of degenerative joint disease in the control group was found to be 4.5%.

In the literature, a limited number of studies investigating the frequency of TMJ involvement in AS patients have reported rates ranging from 4 to 59.2%^14,15,16,17^. In our study, among the AS group consisting of 113 patients, 34 (30.1%) received no diagnosis, while 79 (69.9%) were diagnosed with at least one TMD. Specifically, 43 AS patients (38.1%) had pain disorders and 61 (54%) had joint disorders. In a study published in 2021 by Huang et al.^3^, 3,204 AS patients were compared with a cohort of 12.816 individuals after adjustments for age, gender, and comorbidities. After corrections, the TMD incidence was found to be 2.66 times higher in the AS cohort. In a study by Souza et al.^18^, 30 AS-diagnosed patients were evaluated using Research Diagnostic Criteria for Temporomandibular Disorders (RDC/TMDs) for TMD. Three diagnostic groups were identified: muscle diagnosis, disc displacement, and other conditions (such as osteoarthritis, osteoarthrosis, and arthralgia). Only 1 patient did not belong to any group. Of the remaining 29, 17 (57%) were in all three groups, with disc displacement, particularly with reduction, being the most common. Similarly, in our study, disc displacement with reduction was the most common diagnosis in both the healthy control group and the AS group, as observed in various other studies in the literature^11,12,13^.

In our study, among the diagnosis group of pain disorders, the most frequently observed diagnosis in both the healthy control and AS groups was myalgia, with rates of 20.9% in the healthy group and 37.1% in the AS group. Within the myalgia subgroup, local myalgia was the most common. In the healthy group, arthralgia was observed in 5.4%, and in the AS group, it was observed in 16%. Headache attributed to TMD was found in 4.5% of the healthy group and 9.7% of the AS group. In a study by Alrashdan et al.^13^, myofascial pain was the most frequently observed among pain disorders. Joint disorders were less common compared to pain disorders. However, in our study, joint disorders were more frequently observed than pain disorders in both the healthy and AS patient groups. There are other studies in the literature that, like ours, show that pain disorders are less common compared to joint disorders^11^.

Maximum mouth opening has been reported to be significantly reduced in the AS group compared to the healthy population in many studies^19,20,21,22^. In our study, the AS group had significantly more limited pain-free, maximum assisted, and maximum unassisted opening compared to the healthy group. However, there were no differences in protrusion or lateral movements. Additionally, no significant relationship was found between these measurements and joint or pain disorder diagnoses. In this study, 13 out of 36 individuals (36.1%) with joint disorders in the healthy group had bilateral joint disorders. In the AS group, 37 out of 61 patients (60.7%) had bilateral joint disorders. Alrashdan et al.^13^ found a higher rate of bilateral TMJ involvement in TMJ disorder diagnoses compared to unilateral in their study. However, Iordache et al. found unilateral involvement in 45.4% of their included patients^23^.

In this study, no significant relationship was found between joint disorder diagnoses and BASDAI, BASFI, and BASMI scores. However, a significant relationship was observed between pain disorder diagnoses and BASDAI and BASFI scores. Iordache et al.^23^ found that symptoms and findings related to TMJ in 55 AS patients were associated with BASDAI and BASFI scores. Other studies in the literature have also found correlations between TMJ involvement and disease activity^16,24,25^.

Our study has several strengths and limitations. There are very few studies in the literature that assess the prevalence of TMD in AS patients using DC/TMD criteria. The strengths of our study include the comparative evaluation with a control group and a higher number of patients compared to other studies. However, a limitation is the inability to confirm TMD radiologically.

In conclusion, our study underscores that AS patients exhibit a higher prevalence of TMD diagnoses, pain disorders, and joint disorders compared to healthy individuals. Bilateral joint disorders are particularly prevalent among AS patients. Additionally, a significant relationship was found between the diagnosis of pain disorders and disease activity. Moving forward, objective assessments supported by radiological methods are imperative for advancing research in this field.

## COMPLIANCE WITH ETHICAL STANDARDS

This study obtained all necessary ethical approvals for experiments involving both humans and animals, ensuring that the research was conducted in accordance with established ethical standards. In particular, for human studies, informed consent was diligently obtained from all participants, and their confidentiality rights were strictly upheld.
